# White Bread or Brown Bread?

**Published:** 1899-02

**Authors:** 


### White Bread or Brown Bread?

A recent hospital report by Drs. Lauder Brunton and Tunnicliffe deals with the relative digestibility of white and brown bread. It is thus summarized in *The British Medical Journal,* November 5th: "On the strength of certain experiments, which they describe in full, they feel justified in concluding that the higher nutritive value which might on purely chemical grounds be ascribed to brown bread cannot be maintained from the physiological side. With regard to fats and mineral constituents on the other hand, distinctly less of the nutritive materials actually get into the blood in the case of brown than of white bread. White bread is, weight for weight, more nutritious than brown. It thus would appear that the preference given by operatives in large towns to white bread has, to certain extent, a sound physiological basis. In the case of people with irritable intestines white bread is to be preferred to brown. In the case of people with sluggish bowels brown bread may be preferable to white, as it tends to maintain peristalsis and insures regular evacuation of the bowels. If the proportion of mineral ingredients, and especially of lime salts, in other articles of food or drink be insufficient, brown bread is preferable to white. It is possible that in the case of operatives living chiefly upon bread and tea, the preference for white bread which prevails may be responsible, in part at least, for the early decay of the teeth. . . . Lastly, Drs. Brunton and Tunnicliffe are of opinion that if the dietary be insufficient in fat, or if the patient be unable to digest fat readily in other forms, brown bread may possibly be preferable to white. The authors rightly dwell on the absurdity of taking the mere chemical composition of a foodstuff as an index of its nutritive value. 'A stick of charcoal, the atmospheric air, a little water, and some sea salt, contain all the elements of a typical diet, and in ample quantity.' Hence it is not always a question of what a foodstuff contains, but how it contains it."

